# Global and Specific Profiles of Executive Functioning in Prodromal and Early Psychosis

**DOI:** 10.3389/fpsyt.2019.00356

**Published:** 2019-05-21

**Authors:** Wu Jeong Hwang, Tae Young Lee, Won-Gyo Shin, Minah Kim, Jihyang Kim, Junhee Lee, Jun Soo Kwon

**Affiliations:** ^1^Department of Brain and Cognitive Sciences, College of Natural Science, Seoul National University, Seoul, South Korea; ^2^Department of Psychiatry, College of Medicine, Seoul National University, Seoul, South Korea; ^3^Department of Neuropsychiatry, Seoul National University Hospital, Seoul, South Korea; ^4^Institute of Human Behavioral Medicine, SNU-MRC, Seoul, South Korea

**Keywords:** executive function, psychosis, clinical high risk, neurocognition, semantic fluency, spatial working memory, first-episode psychosis

## Abstract

**Objective:** Numerous reports on neurocognitive functioning deficits in individuals at clinical high risk (CHR) and first-episode psychosis (FEP) patients suggest particular deficits in executive functioning (EF). However, to date, most of the studies have administered a single or a few EF tests to participants, and few investigations have examined the different components of EF to identify specific subdomains of relative strength and weakness.

**Method:** Forty CHR subjects, 85 FEP patients, and 85 healthy controls (HCs) were assessed with a neuropsychological battery to elucidate the profiles of EF in the subdomains of shift, attention, fluency, and planning.

**Results:** In the subdomains of shift, attention, and fluency, CHR individuals and FEP patients showed deficits compared to HC. The *post hoc* analysis revealed that CHR individuals had comparable attention shifting and phonemic fluency compared to FEP. CHR showed intermediate deficits between FEP and HCs in spatial working memory and semantic fluency, and the largest effect size was observed in semantic fluency both for CHR and FEP.

**Conclusion:** Overall, the findings of this study, in addition to providing detailed profiles of EF in prodromal and early psychosis patients, highlight the informative value of the specific subdomains of semantic fluency and spatial working memory.

## Introduction

Impaired cognition across a range of cognitive domains is the hallmark of schizophrenia. Cognitive deficits in attention, learning, memory, and executive functioning (EF) have moderate to large effect sizes of impairments (1). Further, the deficits, especially in domains of attention, processing speed, working memory, verbal declarative memory, and EF are identified not only in chronic schizophrenia patients (2) but also prior to the onset of the disorder (3–5). In fact, distinctive patterns of cognitive deficits at different stages of the disorder exist, and they form before the onset of clinical symptoms, as early as the first episode of psychosis (FEP) (6, 7) or even before the prodromal state (8), the latter of which is also referred to as clinical high risk (CHR) for psychosis (9, 10). The FEP patients show cognitive deficits across on almost all cognitive domains, which are comparable deficits to the fully established disorder, and CHR individuals show intermittent degree of deficits.

Recent efforts have aimed at elucidating neurocognitive deficits prior to psychosis onset in CHR individuals who exhibit clinical features, such as symptoms and behaviors, that place them at increased risk for developing psychosis. To date, studies report small to medium impairments of approximately 0.3–0.6 standard deviations (SDs) below healthy controls (HCs) across various cognitive domains in CHR individuals (11–13) and larger sizes of impairment in FEP patients of 1.0–1.5 SDs below HCs (14–17). However, despite acknowledging the importance of understanding detailed cognitive patterns of CHR individuals and FEP patients, few studies have investigated both groups concurrently. Further, the few articles that exist report huge variations in reported effect sizes, which hinder us from clearly understanding the whole picture. This is most noticeable especially in the domain of EF. Significant factors contributing to this phenomenon are inconsistencies in definition of EF and its subdomains.

Disorders of EF are the most commonly observed cognitive deficits in schizophrenia (18). These disorders may be objectified by neuropsychological tests to examine different aspects of EF. This is because EF refers to complex mental processes that orchestrate purposeful and goal-directed activity that intrinsically underlies almost all of our neuropsychological functioning—verbal fluency, working memory, attention, and planning, to name a few. Further, disturbances in EF lead to impairment in second-order cognitive processes such as memory, language, or emotion, which may eventually produce psychosis symptoms such as hallucinations, negative symptoms, and dysexecutive behaviors (18). A number of reviews and meta-analyses have been conducted, but despite much effort, the current understanding of EF deficits in schizophrenia is limited. This is due to the inconsistencies of EF constructs between studies and the small number of studies evaluating EF across accepted domains of interest. In general, studies investigating EF of psychosis patients have employed a limited range of tasks and yet refer to EF as whole. Further, currently available tests can only capture one subdomain of EF, requiring administration of various tests to obtain a comprehensive view on EF. Hence, the currently available results are speculated to be more related to the variability in difficulty levels of the tests or to the dysfunction degree in the different subfunctions being measured in the investigation group. Thus, there exists criticism of this approach of investigation (19) in favor of moving toward building an overall framework of the EF profile in psychosis.

The consequences of the inconsistencies are best exemplified as discrepancies of EF effect sizes in several review studies. In both groups of FEP and CHR, a range of neurocognitive functions, such as verbal fluency and memory are consistently reported to have large effect sizes. In studies where these functions are grouped under EF, the effect sizes are reported as high (13), whereas in cases where these are classified as separate or are not measured, the effect sizes of EF are reported as low (12). Hence, so far, investigations have led to only crude conclusions that EF of CHR individuals is deficient. Thus, it is necessary to conduct a thorough examination of EF of CHR individuals, FEP patients and HCs, to reveal specific strengths and weaknesses on a subdomain basis that lie in their EF, compared to HCs, and to elucidate the common aspects and distinctive features of EF profiles in CHR individuals and FEP patients.

Therefore, in this study, we examined the EF profile, including all the subdomains of function, of both CHR and FEP individuals who were at different psychosis illness stages and compared their abilities to those of an HC group. We hypothesized that CHR individuals would show poorer performance compared to HCs but better performance compared to FEP patients. The subdomain of 1) shifting was measured by the Trail Making Test (TMT) and Wisconsin Card Sorting Test (WCST), 2) attention was measured by the Stop Signaling Test (SST), 3) fluency was measured by the Controlled Oral Word Association (COWA) test, and 4) planning was measured by the Rey–Osterrieth Complex Figure Test (RCFT) and Spatial Working Memory (SWM) Test.

## Methods

### Participants

Forty subjects at CHR, 85 FEP patients, and 85 HCs who participated in the prospective and longitudinal high-risk cohort study conducted at the Seoul Youth Clinic were involved in this research (20). All participants made initial contact with the Seoul Youth Clinic by telephone, by website (http://www.youthclinic.org), or by a referral from a local clinic.

Rigorous clinical interviews were administered to all FEP and CHR individuals by experienced psychiatrists using the Structured Clinical Interview for Diagnostic and statistical manual of mental disorders (DSM-IV) Axis I (SCID-I) disorders to identify past and current psychiatric illnesses. For FEP, inclusion criteria were having schizophreniform disorder, schizophrenia, or schizoaffective disorder in accordance with the DSM-IV criteria with a duration of symptoms of less than 2 years. At the time of assessment, 77.6% (*n* = 66) were receiving atypical antipsychotic medication, with a mean olanzapine-equivalent dose of 10.1 mg/day (SD = 11.3 mg/day), and 19 were not receiving any antipsychotic medication.

The CHR individuals were administered the validated Korean version of the Structured Interview of Prodromal Symptoms (SIPS). To be included, they had to fulfill at least one of the three established criteria for prodromal psychosis state: attenuated positive symptoms, brief intermittent psychotic symptoms (BIPS) below the threshold required for a DSM-IV Axis I psychotic disorder diagnosis, or a 30% decline in global functioning over the past year as well as a diagnosis of schizotypal personality disorder or a first-degree relative with psychosis.

HCs were recruited through an Internet advertisement. Exclusion criteria for HCs included past or current SCID-I Non-Patient Edition (SCID-NP) axis I diagnoses and any first- to third-degree biological relative with a psychotic disorder. The common exclusion criteria for all participants are as follows: substance use disorder, neurological disease, significant head injury accompanying loss of consciousness, evidence of significant medical illnesses that could manifest as psychiatric symptoms, and intellectual disability (IQ < 70). Informed consent was obtained from all subjects, in writing, and the study was conducted in accordance with the Declaration of Helsinki. The study was also approved by the Institutional Review Board of the Seoul National University Hospital.

### Clinical and Neurocognitive Function Assessments

The Positive and Negative Syndrome Scale (PANSS) and the Global Assessment of Functioning (GAF) were administered to both CHR and FEP groups. To estimate each subject’s IQ, the Korean version of the Wechsler Adult Intelligence Scale (K-WAIS) was administered.

To assess EF and its subdomains, the following neuropsychological tasks were administered: 1) To assess participants’ attention shifting, the TMT (21) Part A and Part B were administered, and scores from Part A were subtracted from Part B, which enabled acquisition of the TMT B-A score. 2) Participants’ cognitive flexibility was measured by administering the WCST (22), in which the number of perseverative responses was calculated. 3) Response inhibition was evaluated with the SST (23), for which we calculated the stop signal reaction time (SST SSRT). 4) A verbal fluency test, the COWA (24), was administered. It measured the spontaneous oral generation of words within a fixed time span based on phonemic (phonological fluency, COWA Word) or semantic criteria (semantic fluency, COWA Category). 5) To evaluate participants’ visual memory, the RCFT (25), in which we calculated immediate and delayed scores and organization strategy scores, was given. 6) Participants’ working memory was assessed by administering the SWM (26), in which the error scores were calculated (see **Table 1**).

**Table 1:** Description of the tasks.

Test	Description	Outcome	Executive function subdomain
TMT B-A	Part B (the time taken to connect consecutively numbers and letters, alternating between them); Part A (the time taken to connect numbers in consecutive order)	Total time for completion, in seconds	Shifting (Attention shifting)
WCST Perseveration	To sort cards according to a rule that the participant has to figure, and after a run of trials, the rule is changed without warning	Number of errors where the participant has applied the same rule for their choice as the previous choice	Shifting (Cognitive flexibility)
SWM Error	To find a token in each of a number of boxes and the number of boxes increases with the task progression	Number of times the subject revisits a box in which a token has previously been found	Planning (Working memory)
SST SSRT	To respond to an arrow stimulus and to withhold making the response upon an auditory signal	Stop signal reaction time	Attention (Response inhibition)
RCFT Planning	To reproduce a complicated line drawing, first by copying it freehand, and then by drawing from memory.	Points given for constructing each configural segment as an unfragmented unit	Planning
COWA Word	To say all the words that they can that begin with a given letter	Number of correct words	Fluency (Phonological)
COWA Category	To say all the words that they can that belong to a given category	Number of correct words	Fluency (Semantic)

TMT B-A, Trail Making Test Part B–Part A; WCST, Wisconsin Card Sorting Test; SWM, Spatial Working Memory; SST, Stop Signaling Test; SSRT, stop signal reaction time; RCFT, Rey–Osterrieth Complex Figure Test; COWA, Controlled Oral Association Test.

### Statistical Analysis

Data analysis was conducted using Statistical package for the social sciences (SPSS) V.24.0 (SPSS Inc., 2016; PC version). Neuropsychological variables were assessed for normality (skewing and kurtosis). The test scores were standardized to the performance of the control group (*z* scored), and error scores were sign-changed to provide a standard metric for comparison across tests. A series of univariate analysis of covariance (ANCOVA) tests was conducted to examine differences in EF subdomain performances, with group (CHR, FEP, and HCs) as a between-participant factor and test performance scores as dependent variables. Furthermore, the demographic variables of sex and olanzapine-equivalent doses, which were significantly different between groups, were included as covariates. To detail group differences, *post hoc* Bonferroni-corrected pairwise comparisons were used. Multiple testing was controlled by the stepdown Bonferroni–Holm procedure (starting alpha level 0.05/7 = 0.007). Descriptive statistics for these variables are shown in **Table 2**. Effect sizes are reported as Cohen’s *d*.

**Table 2:** Demographic and clinical characteristics of the subjects.

Variables		CHR	FEP	HCs	χ^2^, *F*, or *T*	*P*
		(*n* = 40)	(*n* = 85)	(*n* = 85)		
Age (y)		20.55 ± 3.05	21.87 ± 3.58	21.24 ± 2.35	2.70	.069
Sex (M/F)		28/12	40/45	51/34	3.29	.039*
IQ		108.30 ± 11.65	100.27 ± 15.66	111.95 ± 13.53	14.93	<.001*
Education (y)		12.98 ± 1.23	13.42 ± 2.06	13.73 ± 1.25	2.99	.053
Parental SES		2.83 ± .98	2.65 ± .84	2.62 ± .76	0.83	.440
PANSS	Total	NA	62.38 ± 13.30	NA	−2.00	.080
	Positive	NA	13.32 ± 2.81	NA	−4.08	.001*
	Negative	NA	176.44 ± 5.65	NA	−0.68	.519
	General	NA	32.72 ± 7.62	NA	−0.95	.347
SOPS	Total	35.55 ± 1.73	NA	NA	NA	NA
	Positive	9.63 ± 0.58	NA	NA	NA	NA
	Negative	14.75 ± 0.93	NA	NA	NA	NA
	General	6.83 ± 0.61	MA	NA	NA	NA
	Disorganization	4.36 ± 0.43	NA	NA	NA	NA
GAF		50.68 ± 7.31	50.68 ± 7.31	NA	1.81	.104
Duration of illness (years)		2.35 ± 1.75	0.54 ± 0.52	NA	NA	NA
Olanzapine-equivalent dose (mg/day)		NA	10.12 ± 11.29	NA	NA	NA

FEP, first-episode psychosis; CHR, clinical high risk for psychosis; HCs, healthy controls; GAF, Global Assessment of Functioning; PANSS, Positive and Negative Syndrome Scale; SES, socioeconomic status. Data are given as the mean ± standard deviation.

*Significant results are presented as: *P < .05.

## Results

### Demographic and Clinical Characteristics

There were no significant differences in age, years of education, or parental socioeconomic status in the participating individuals. Both the CHR and FEP groups had a significantly lower current IQ than the control sample, and FEP had a lower current IQ than CHR (F_2,207_ = 22.59, *p* < .001). Significant differences were also found in the demographic variable of sex: there were more females in the FEP than CHR and HC groups (F_2,207_ = 9.68, *p* < .01) (**Table 2**).

### Neuropsychological Functioning Tests

Each of the ANCOVAs demonstrated a significant main effect of group (Attention: F_2,202_ = 9.092, *p* < .001; Shifting: F_4,404_ = 10.5, *p* < .001; Fluency: F_4,404_ = 10.169, *p* < .001; Planning: F_4,404_ = 11.526, *p* < .001) except for RCFT (**Table 3**).

**Table 3:** Means of raw scores or scaled scores of CHR, FEP, and HCs for each of the executive function domains.

Neurocognitive task	CHR (*n* = 40)	FEP (*n* = 85)	HCs (*n* = 85)	*F* (2,205)	*p*	CHR	FEP	Comparisons
	Mean (SD)	Mean (SD)	Mean (SD)			d^†^	d^†^	
Shifting							
TMT B-A	−45.72 (23.73)	−58.54 (47.47)	−31.8 (14.79)	18.47	<.001*	.53	.57	HC > FEP, CHR > FEP
WCST Perseveration	−11.89 (7.00)	−15.84 (11.15)	−9.84 (5.78)	13.83	<.001*	.33	.47	HC > FEP
Attention							
SST SSRT	−215.16 (104.89)	−228.69 (117.58)	−173.03 (65.58)	9.48	<.001*	.32	.40	HC > FEP
Fluency							
COWA Word	35.94 (7.90)	31.31 (8.34)	41.33 (8.48)	41.42	<.001*	.41	.77	HC > FEP, CHR > FEP
COWA Category	39.09 (10.10)	32.31 (11.93)	45.50 (10.58)	39.97	<.001*	.64	.85	HC > CHR > FEP
Planning							
RCFT Planning	3.00 (0.71)	3.25 (0.84)	3.27 (0.76)	2.36	0.189			
SWM Errors	−20.40 (18.08)	−28.12 (22.14)	−11.75 (12.26)	21.12	<.001*	.56	.62	HC > CHR > FEP

SD, standard deviation.

^†^Effect size calculated by Cohen’s d.

*Significant at an alpha level of .007, adjusted for sex and olanzapine-equivalent dose.

**Figure 1:**
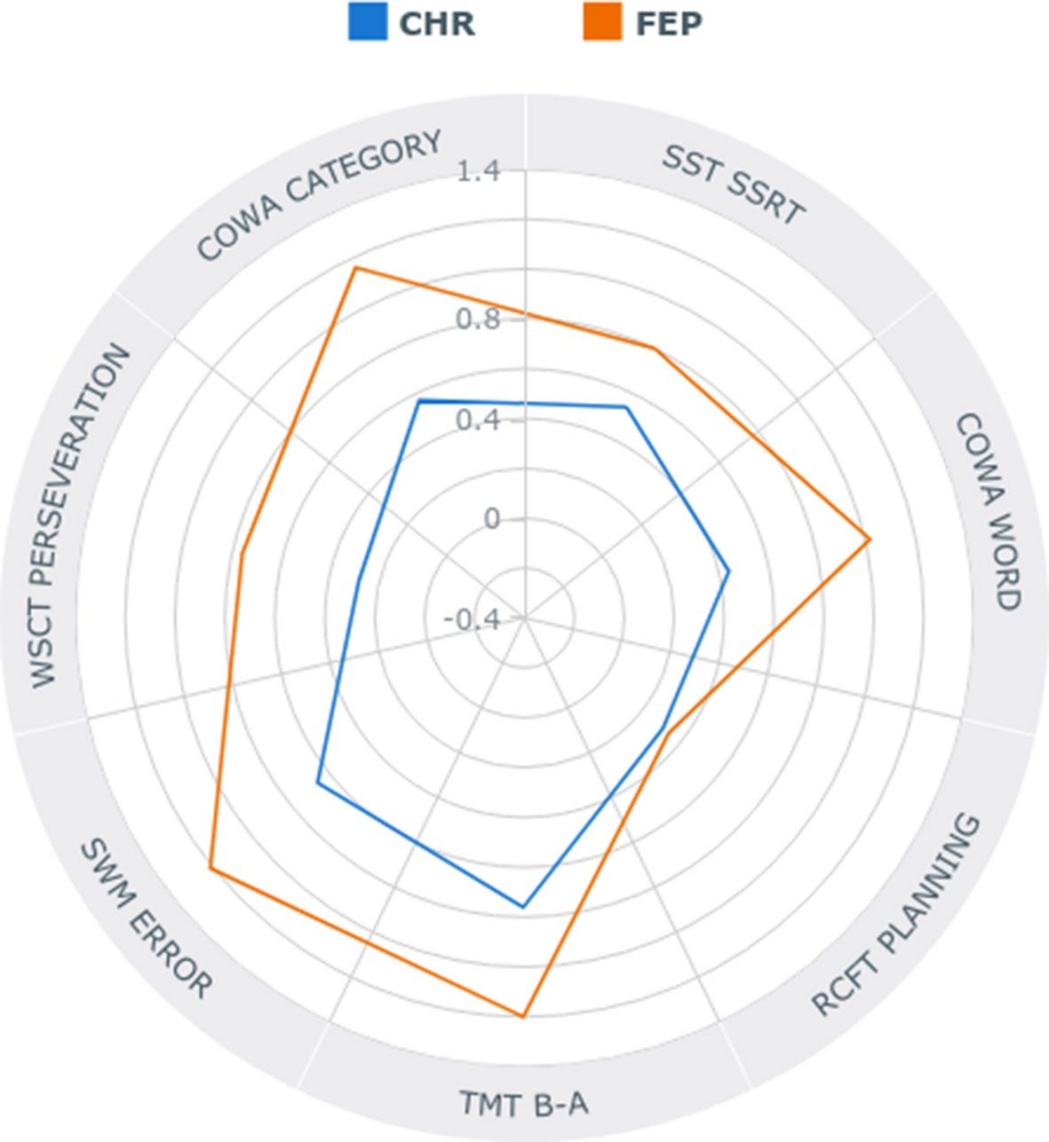
Radar plot showing specific profiles of executive functioning in individuals at clinical high risk (CHR) for psychosis and first-episode psychosis (FEP) in absolute *z*-scores.

Follow-up analyses on the subdomain of shifting showed higher abilities of HCs compared to FEP in attention shifting (*p* < .001) and cognitive flexibility (*p* < .001) measured by subtracting the TMT-A score from the TMT-B scores and by the WCST perseveration score, respectively. CHR individuals showed better ability to shift their attention compared to FEP (*p* = .01). Impaired performance of inhibiting responses, compared to HCs, was observed in FEP (*p* < .001). Furthermore, *post hoc* analyses on fluency showed FEP had the most impaired semantic word fluency, with CHR having an intermediate level between the FEP and HCs. However, for phonemic fluency, both HCs and CHR were better than FEP (*p* < .001; *p* = .037, respectively). Lastly, for the planning subdomain, CHR showed intermediate spatial working memory ability, with HCs showing the best and FEP showing the worst (**Figure 1**). For Z-score means of each EF domain for CHR individuals and FEP patients, please refer to **Supplementary Table 1**.

### Effect Size Analysis

**Table 3** lists effect sizes as Cohen’s *d* corresponding to those group differences that remained significant after adjusting for multiple testing. The FEP and CHR groups demonstrated performance deficits with respective effect sizes from 0.4 and 0.32 (inhibitory control of attention) to 0.62 and 0.56 (spatial working memory) and to 0.85 and 0.64 (semantic fluency) compared with HCs (**Table 3**).

### Addition of IQ as a Covariate

**Supplementary Table 2** describes the results when IQ was added as a covariate in our analysis, as well as sex and olanzapine-equivalent dose.

## Discussion

The present study examined the subdomains of EF in CHR and FEP to elucidate the patterns of EF profiles. The findings, in line with other studies, suggest broad impairment of EF performance in early psychotic individuals and individuals at risk for psychosis. However, despite showing similar patterns of deficits at a subdomain level, when each functioning within each subdomain was examined, different patterns were revealed between prodromal and early psychosis, with variable effect sizes. Gradual impairments, from prodromal to early psychosis, were observed in spatial working memory and semantic fluency. The latter functioning also had the largest effect sizes in both groups, suggesting it as a highly sensitive functioning for detection of both CHR individuals and FEP patients. Attention shifting and phonemic fluency were impaired in FEP patients but comparable in CHR individuals. Further, the integrities of cognitive flexibility and response inhibition were not detected for CHR individuals. Overall, the findings of this study highlight the informative values and the detailed nature of EF in early psychosis and individuals at risk for psychosis.

Abundant literature reveals substantial impairments in a range of cognitive functions in FEP patients (8, 19, 27, 28). In line with the literature, we also found broad impairments of EF in FEP patients in areas of attention shifting, cognitive flexibility, inhibition of attention, spatial working memory, and fluencies in phonemics and semantics (8, 29). Unlike the current literature, we did not find a significant deficit in the planning ability of FEP patients (30, 31). We cautiously speculate that this was due to the sensitivity of the test utilized in this study. Whereas the past studies (30, 31) have administered the Tower of London or modified versions of it to measure planning deficits of FEP patients, we utilized the planning score derived from RCFT. Although RCFT is a valid measure of one’s visuospatial abilities, organizing skills, and planning abilities, most studies in the field of psychosis research have utilized its measures of copy and recall (32–34), rather than planning. Although the planning measure is indeed a reliable and sensitive measure in the research field of obsessive-compulsive disorders (35), the results of our current study lead us to cautiously speculate that the planning measure of RCFT may not be sensitive enough to detect planning deficits in FEP patients or may capture different aspects of planning than the Tower of London test.

The CHR individuals showed fluctuations in their EF profiles, and there were three distinctive patterns: 1) comparable abilities to HCs in attention shifting and phonemic fluency; 2) significant deficits compared to HCs but still outperforming FEP patients in semantic fluency and spatial working memory; and 3) no statistically evident detection of significant deficits or preservation in cognitive flexibility or inhibition of attention. This pattern of fluctuations, as well as elucidating the strengths and weaknesses of EF function in CHR individuals, is evidence that there exist areas where currently employed tests fail to detect the subtle deficits.

We found significantly comparable ability of phonemic fluency in CHR individuals. Indeed, this is one of the most consistent findings in cognition studies of CHR individuals (36–38). Further, we found significantly disturbed semantic fluency in the same group. This phenomenon of comparable phonemic fluency but disturbed semantic fluency is one of the consistent findings in cognition studies of CHR individuals (36–38). This is thought to be due to the different underlying mechanisms involved in retrieving the stored information. While semantic fluency highly depends on activation flow through the semantic network, phonemic fluency depends on search and retrieval from the lexicon using phonemic or orthographic cues. Furthermore, on a brain-circuit level, unlike phonological processing, which requires activation of a number of fontal and temporal sites, semantic processing requires middle and superior temporal sites (39), and semantic processing involves a high degree of interhemispheric connectivity (40). Evidence suggests frontal bilaterality in verbal fluency in CHR individuals (41), and their semantic fluency deficit may be the result of the failure of lateralization (38), upon which semantic fluency partly depends. Further investigation is required to elucidate the similarities and differences in the neural mechanisms underlying semantic and phonemic fluency in psychosis patients.

Currently, semantic fluency deficit is considered as a candidate trait marker in schizophrenia. A meta-analysis by Szoke et al. (42) that investigated longitudinal studies of cognitive performance of schizophrenia patients showed that, unlike phonological fluency, semantic fluency remained stable, suggesting it is a persistent cognitive deficit that may be considered a potential trait marker in schizophrenia. Our results showing altered semantic fluency in CHR subjects and FEP patients further support this notion, together with the literature findings reporting deficits in first-degree relatives and the stability of the aforementioned results in schizophrenia patients (43, 44). Further, in a longitudinal study design for 2 years, we have previously reported persistent semantic fluency deficits in CHR subjects (45). To add more, in a recent meta-analysis by Fusar-Poli et al. (12), a range of neuropsychological functions was investigated in CHR individuals, and verbal fluency functioning, as well as working memory, was reported as one of the factors associated with the transition to psychosis, suggesting that it may be useful for early intervention.

We found significant deficits of spatial working memory in CHR individuals and FEP patients compared to HCs, with CHR individuals having significantly better performance than FEP patients. Indeed, the deficit in visuospatial working memory is fundamental to schizophrenia (46). Mounting evidence exists for spatial working memory deficits in CHR individuals (12), FEP patients (47, 48), people at genetic risk for schizophrenia (49), and individuals with schizotypal personality disorder (50), suggesting its function as a cognitive marker of an increased vulnerability to disease. In a recent meta-analysis by Fusar-Poli et al. (12), CHR individuals had impaired working memory compared to controls, with an effect size of 0.36, and the CHR individuals who subsequently transitioned to psychosis had poorer working memory than the individuals who did not. However, there also exists evidence otherwise, and authors have failed to see spatial working memory as a possible indicator of psychosis onset (51). Functional neuroimaging studies also report altered regional brain activation during working memory performance in CHR individuals compared to controls, but similar to the current study’s pattern, it was to a lesser degree than in FEP patients (52–54).

Our findings should be interpreted in the context of several limitations. First, the planning factor was based upon errors in planning ability. In future studies, it would be beneficial to examine specific aspects of planning to tease apart the components of planning (e.g., formation versus execution of a plan). Furthermore, additional measures of planning exist (e.g., Tower of London) that may reveal planning abilities in other contexts. Similarly, additional measures, such as sustained attention, divided attention, and selective attention, could be employed for the attention factor to capture specific attentional deficits in psychosis patients. Second, this study, being a cross-sectional study, cannot account for the potential variability that may be present within individuals and does not suggest direct evidence that EF functions decline with the onset of psychosis or that the psychosis onset affects EF functions. Longitudinal studies suggest no cognitive decline from the psychosis prodrome to the FEP (8, 55, 56) and no associations between EF and psychotic (57). Further, we have not collected the data on how many patients declined participation. Thus, there may be potential recruitment bias. Lastly, different cognitive types of CHR or FEP may be present. Studies have reported the existence of different cognitive subtypes in CHR and FEP (2, 58–61). For example, we have reported baseline differences in neurocognitive functioning between remitting and nonremitting CHR individuals in a longitudinal study. The CHR nonconverters who later remitted did not show any significant baseline cognitive deficits compared to HCs (62).

The current study, as well as suggesting EF requires a thorough examination in individuals at different stages of psychosis illness, also highlights the significance of examining different components of EF to identify specific subdomains of relative strength and weakness, rather than administering a single or a few tests and summarizing that EF is globally intact or deficient. Further, this approach can be taken to a global level, when applied across multiple psychiatric disorders, and be utilized to provide evidence of disorder-specific profiles, which duly supports the current movement toward a shared-assessment approach. The clinicians, then, may be able to select tests that are most sensitive to disorder-specific patterns. The present findings of individuals at different psychosis illness stages indicate that semantic fluency and spatial working memory can be utilized to distinguish individuals at different stages of psychosis. Overall, the findings of this study highlight the informative value and the detailed nature of EF in individuals with early psychosis and individuals at risk for psychosis, especially in the subdomains of semantic fluency and spatial working memory.

## Ethics Statement

This study was carried out in accordance with the recommendations of the Seoul National University Hospital Institutional Review Board with written informed consent from all subjects. All subjects gave written informed consent in accordance with the Declaration of Helsinki. The protocol was approved by the Seoul National University Hospital Institutional Review Board.

## Author Contributions

WH contributed to performing the literature search, data analysis, interpretation of the results and to drafting the manuscript. TL and JK contributed to the study design, analysis plans and to editing the manuscript. JL contributed to collecting the clinical information of the participants. JK contributed to the data collection and data analysis. WS contributed to the data collection, analysis plan and to editing the manuscript. MK contributed to the editing the manuscript.

## Funding

The authors disclose receipt of the following financial support for the research, authorship, and/or publication of this article: This research was supported by the Brain Research Program through the National Research Foundation of Korea (NRF), funded by the Ministry of Science, ICT & Future Planning (grant no. 2017M3C7A1029610).

## Conflict of Interest Statement

The authors declare that the research was conducted in the absence of any commercial or financial relationships that could be construed as a potential conflict of interest.
