# Supplementary material for: Global and Specific Profiles of Executive Functioning in Prodromal and Early Psychosis
Source: Front Psychiatry. 2019 May 21;10:356. doi: 10.3389/fpsyt.2019.00356 (PMC6537881; doi:10.3389/fpsyt.2019.00356)
Supplement: Supplementary file 1 [file Table_1.docx]

Global and Specific Profiles of Executive Functioning in Prodromal and Early Psychosis

***Supplemental Information***

**Supplementary Table 1: Z-score means of Executive Function Domains
for CHR individuals and FEP patients**

|  | CHR (n = 40) | FEP (n = 85) |
| --- | --- | --- |
| Neurocognitive Task | Mean (SD) | Mean (SD) |
|  |  |  |
| **Shifting** |  |  |
| TMT B-A | 0.76 (1.45) | 1.20 (2.09) |
| WCST Perseveration | 0.28 (1.27) | 0.76 (1.61) |
| **Attention** |  |  |
| SST SSRT | 0.54 (1.50) | 0.80 (1.67) |
| **Fluency** |  |  |
| COWA Word | 0.44 (0.88) | 1.02 (1.14) |
| COWA Category | 0.57 (0.99) | 1.16 (0.98) |
| **Planning** |  |  |
| RCFT Planning | 0.31 (0.96) | 0.34 (1.24) |
| SWM Errors | 0.66 (1.53) | 1.21 (1.67) |

CHR, Clinical-High Risk for Psychosis; FEP, First-Episode Psychosis; TMT, Trail
Making Test; WCST, Wisconsin Card Sorting Test; SST, Stop Signal Test; COWA,
Controlled Oral Word Association Test; RCFT, Rey-Complex Figure Test, SWM,
Spatial Working Memory Test; SD, standard deviation.

**Addition of IQ as a covariate**

**Methods**

Data analysis was conducted using SPSS V.24.0 (SPSS Inc., 2016; PC version). Neuropsychological variables were assessed for normality (skewing and kurtosis). The test scores were standardized to the performance of the control group (z scored), and error scores were sign-changed to provide a standard metric for comparison across tests. A series of univariate analysis of covariance (ANCOVA) tests was conducted to examine differences in EF subdomain performances, with group (CHR, FEP and HCs) as a between-participant factor and test performance scores as dependent variables. Furthermore, the demographic variables of sex, IQ and olanzapine-equivalent doses, which were significantly different between groups, were included as covariates. To detail group differences, *post hoc* Bonferroni-corrected pairwise comparisons were used. Multiple testing was controlled by the stepdown Bonferroni-Holms procedure (starting alpha level 0.05/7=0.007).

**Results**

**Supplementary Table 2.** **Means of Raw Scores or Scaled Scores of CHR, FEP and HCs for
Executive Function Domains with covariates of IQ, sex and olanzapine-equivalent dose**

|  | CHR (n = 40) | FEP (n = 85) | HCs (n = 85) |  |  |  |
| --- | --- | --- | --- | --- | --- | --- |
| Neurocognitive Task | Mean (SD) | Mean (SD) | Mean (SD) | F (2,204) | p | Post hoc |
|  |  |  |  |  |  | comparisons |
| **Shifting** |  |  |  |  |  |  |
| TMT B-A | -45.72 (23.73) | -58.54 (47.47) | -31.8 (14.79) | 9.99 | <.001* | HC>FEP, |
| WCST Perseveration | -11.89 (7.00) | -15.84 (11.15) | -9.84 (5.78) | 4.31 | <.015 | HC>FEP |
| **Attention** |  |  |  |  |  |  |
| SST SSRT | -215.16 (104.89) | -228.69 (117.58) | -173.03 (65.58) | 2.73 | <.067 | HC>FEP |
| **Fluency** |  |  |  |  |  |  |
| COWA Word | 35.94 (7.90) | 31.31 (8.34) | 41.33 (8.48) | 8.44 | <.001* | HC>FEP |
| COWA Category | 39.09 (10.10) | 32.31 (11.93) | 45.50 (10.58) | 11.08 | <.001* | HC>FEP |
| **Planning** |  |  |  |  |  |  |
| RCFT Planning | 3.00 (0.71) | 3.25 (0.84) | 3.27 (0.76) | 1.34 | 0.264 |  |
| SWM Errors | -20.40 (18.08) | -28.12 (22.14) | -11.75 (12.26) | 15.37 | <.001* | HC>FEP, CHR>FEP |

FEP, first-episode psychosis; CHR, clinical high risk; HCs, healthy controls; TMT, Trail Making Test; WCST,
Wisconsin Card Sorting Test; SST, Stop Signal Test; COWA, Controlled Oral Word Association Test; RCFT,
Rey-Complex Figure Test, SWM, Spatial Working Memory Test; SD, standard deviation.
*Significant at an alpha level of .007, adjusted for IQ, sex and olanzapine-equivalent dose.

**Discussion**

Variety of factors, such as the number of education years, age, sex, antipsychotics and IQ, are associated with executive functioning (EF). However, less investigations are present exploring their associations with EF, particularly, in the clinical population. Often, it is due to some factors being a characteristic feature of the disorder for which cannot be controlled. Individuals at clinical high risk for psychosis (CHR), who are at the prodromal phase of psychosis, are younger, thus having less number of education years than first-episode psychosis (FEP) patients, and these are their natural features that cannot be controlled. Further, lower IQ compared to healthy is a feature of FEP patients, the phenomenon of which is manifested by their general cognitive symptom. However, the problems in inclusion of IQ as a covariate to cognitive studies have been addressed in multiple cognition studies in psychosis (Bora et al., 2015, Fusar-Poli et al., 2012). Evidence of a relationship of IQ to EF is not quite strong and there exist inconsistencies in the results. Studies suggest it is due to several factors, such as differential effects being present on different EF domains (Friedman et al., (2006)), ceiling effects of some tests (Waldmann, Dickson, Monahan, & Kazelskis, 1992) and restricted IQ ranges (Wiens & Matazzaro, 1988). Nonetheless, the relationship of IQ to EF requires deeper investigation and we conducted further analysis to explore the association. The results suggest, in CHR individuals, that their deficits, such as those found in semantic fluency and spatial working memory, may be associated with their IQ. However, unlike spatial working memory, the *posthoc* analysis could not reveal whether there is a deficit or intact ability of semantic fluency in CHR individuals. Further, the CHR individuals of our study do not have lower IQ than HCs. Thus, in this analysis, we cautiously state that the executive function of CHR individuals could have been over-adjusted for their IQ. Further, the degree to which IQ effects EF cannot be addressed with the current study design and analytic method thus, it needs to be investigated in future studies.

**Reference**

1. Bora E, Murray RM. Meta-analysis of cognitive deficits in ultra-high risk to psychosis and first-episode psychosis: do the cognitive deficits progress over, or after, the onset of psychosis? Schizophr Bull. 2014 Jul;40(4):744-755.

2. Fusar-Poli P, Deste G, Smieskova R, Barlati S, Yung AR, Howes O, Stieglitz RD, Vita A, McGuire P, Borgwardt S. Cognitive functioning in prodromal psychosis: a meta-analysis. Arch Gen Psychiatry. 2012 Jun;69(6):562-571.

3. Friedman, N. P., Miyake, A., Corley, R. P., Young, S. E., DeFries, J. C., & Hewitt, J. K. (2006). Not all executive functions are related to intelligence. Psychological Science, 17(2), 172–179.

4. Waldmann, B. W., Dickson, A. L., Monahan, M. C., & Kazelskis, R. (1992). The relationship between intellectual ability and adult performance on the Trail Making Test and the Symbol Digit Modalities. Journal of Clinical Psychology, 48(3), 360–363.

5. Wiens, A. N., & Matazzaro, J. D. (1988). WAIS and MMPI correlates of the Halstead–Reitan neuropsychological battery in normal male subjects. Journal of Nervous and Mental Diseases, 164, 112–123.
